# A global dataset of experimental intercropping and agroforestry studies in horticulture

**DOI:** 10.1038/s41597-023-02831-7

**Published:** 2024-01-02

**Authors:** Raphaël Paut, Léa Garreau, Guillaume Ollivier, Rodolphe Sabatier, Marc Tchamitchian

**Affiliations:** 1 Université Paris-Saclay, UMR Agronomie, INRAE, AgroParisTech, 91123 Palaiseau, France; 2 ECODEVELOPPEMENT, INRAE, 84000 Avignon, France

**Keywords:** Agroecology, Plant ecology

## Abstract

Intercropping and agroforestry systems have been increasingly well studied and documented. Yet, so far, no dataset has provided a systematic synthesis of existing data on intercropping experiments in the specific field of horticulture. A systematic literature search was carried using search terms and applied to Web of Science. The resulting dataset includes data from field experiments published in 191 articles covering experiments worldwide, between 1982 and 2022. The selected experiments cover five continents and involved 118 different crop species. Through manual extraction of information from publications, the dataset includes (i) general information on the articles; (ii) experimental site soil and climate conditions; (iii) descriptions of intercropping designs; (iv) crop management practices; (v) measurements of sole crop and intercrop yields and (v) Land Equivalent Ratios. The dataset is arranged in an easily reusable spreadsheet with columns as variables (n = 45) and rows as treatment (n = 1544). The dataset is freely reusable and updateable. We expect that it will provide valuable information for statistical analysis, modeling and innovative farming system design based on intercropping.

## Background & Summary

In the context of global changes, current agriculture is facing major challenges. With stagnating yields^1^, declining agricultural land area, and a growing food demand^2^, world food systems will have to find new ways to increase production^3,4^. In addition, environmental issues are becoming increasingly important, and expanding biofuel use will dramatically increase the pressure on global agriculture^5,6^. In short, we need to produce more, in a more ecological way, and with less available land, which would imply major changes in the way we produce and consume food.

In this context, intercropping (i.e. the simultaneous growth of two or more crops in the same field area) and agroforestry systems (i.e. the simultaneous growth of trees and crops) have gained attention worldwide and appear to be a promising model of ecological intensification to produce more with lower environmental impact^7,8^. Research has often highlighted the benefits of intercropping and agroforestry systems (IAS) for their positive effects on productivity^9,10^, better use of biotic and abiotic resources^11–13^, enhancing soil fertility and nutrient cycling^14^, or controlling pests and diseases^15,16^. In the specific case of horticulture, defined here as including fruit and vegetable crops, data on the effects of crop association is however missing. Indeed, given the high variety of crop species compared to other production systems, the number of possible intercropping systems is exponential, while research studies remain patchy and heterogeneous.

Numerous field trials conducted over the past 40 years have evaluated the agronomic performance of intercropping systems including horticultural species. Depending on the choice of species, climatic factors, soil types or management practices, these performances differ between sites and growing seasons. Yet, so far, no existing dataset has provided a systematic synthesis of existing data on IAS experiments in the specific field of horticulture. Providing agronomic scientists with such a dataset on IAS over a wide range of environments would make it possible to assess intercrops regarding their capacities to maintain and improve the productivity of agricultural land.

**Fig. 1:**
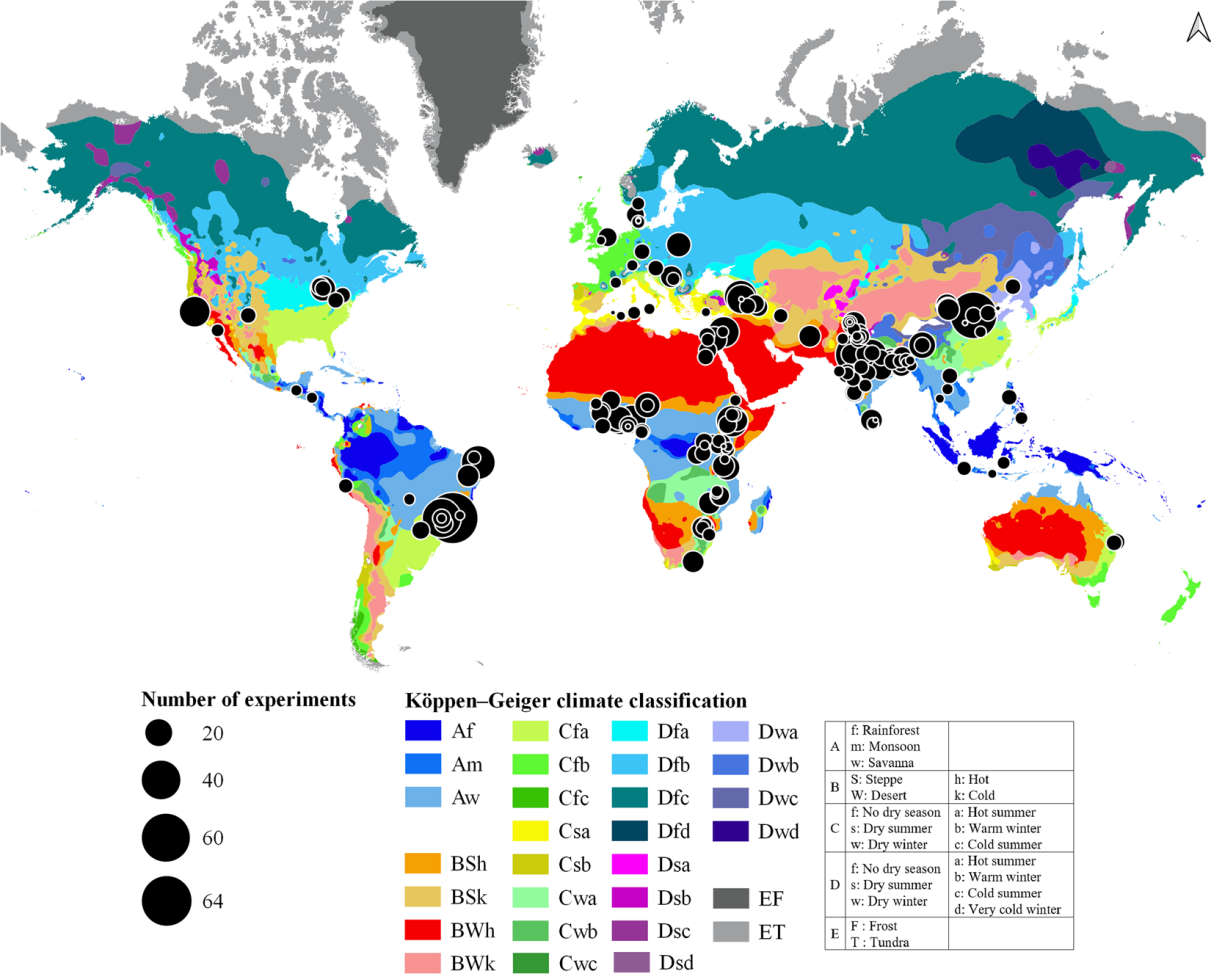
Geographical distribution of sites and number of experiments included in the database. The Köppen-Geiger climatic classification was used to link each field site to a grid size with a resolution of 0.50 degrees of latitude by 0.50 degrees of longitude. The five main Köppen-Geiger climatic zones are represented by acronyms beginning with the letter A (tropical), B (arid), C (temperate), D (continental), and E (polar). Within each climatic zone, each Köppen-Geiger climatic subzone is indicated by a color gradient.

In this paper, we present a global dataset based on results from field intercropping experiments including 118 crop species worldwide, established between 1982 and 2022. Experimental data were extracted from 191 published articles. In total, 1544 experiments were collected across 19 Köppen-Geiger^17^ climatic zones in 45 countries over five continents (Fig. 1). Through manual extraction of information from publications, the dataset includes (i) general information on the articles; (ii) experimental site soil and climate conditions; (iii) descriptions of intercropping designs; (iv) crop management practices; (v) measurements of sole crop and intercrop yields and (v) Land Equivalent Ratios (Table 1).

**Table 1:** Extract from the description and definition of variables included in the dataset.

Group of variables	Variables	Definition	Type	Units/categories	Total number (percentage) of available data
Experimental site soil and climate conditions	Experiment_period	The period over which the experiment was conducted (*e.g*. 2003–2006)	Continuous	—	1504 (97.4%)
	Experiment_year	The year of a specific treatment *(e.g. 2005)*	Continuous	—	1486 (96.2%)
	Country	Country where experiments were carried out	—	—	1544 (100%)
	Study_site	Study site where experiments were carried out	—	—	1544 (100%)
	Latitude and longitude	Latitude and longitude of experimental site	Continuous	Decimal degrees	1538 (99.6%)
	Climate_zone	Climate zone according to Koppen Geiger classification	Categorical	From Koppen-Geiger classification	1534 (99.4%)
	Soil texture	Soil texture recoded according to the USDA soil texture calculator	Categorical	—	900 (58.3%)
	Soil_pH	Soil pH	Continuous	—	871 (56.4%)
Crop management practices	Greenhouse	Greenhouse conditions	Binary	Yes/No	1340 (86.8%)
	Organic_ferti	Use of organic fertilizer	Binary	Yes/No	1113 (72.1%)
	Mineral_ferti	Use of mineral fertilizer	Binary	Yes/No	1309 (84.8%)
	Nitrogen_rate	Nitrogen rate	Continuous	Kg N ha^−1^	1222 (79.1%)
	Pesticide	Use of pesticides	Binary	Yes/No	537 (34.8%)
	Irrigation	Use of irrigation	Binary	Yes/No	730 (47.3%)
Intercropping design descriptors	Intercropping_design	In which design both species were intercropped	Categorical	Replacement, Additive	1258 (81.5%)
	Intercropping_pattern	In which way both species were spatially sown	Categorical	Strip, row, mixed, agroforestry (AF)	1337 (86.6%)
Crop and yield variables	Crop_X_Scientific_Name and Crop_X_Common_Name	Scientific and common names of intercropped species *X*, according to USDA Plant Database	—	From USDA Plants Database	3088 (100%)
	Crop_X_yield_sole	Yield of crop *X* as sole crop	Continuous	—	2242 (72.6%)
	Crop_X_yield_intercropped	Yield of crop *X* in intercropping	Continuous	—	2402 (77.8%)
	Yield_total_intercropping	Total yield of the intercropping system	Continuous	—	1078 (69.8%)
	Yield_measure	Indicates what type of yield was recorded (e.g. grain yield, dry weight, marketable yield, etc.)	Categorical	—	1016 (65.8%)
	LER_crop_X	Partial Land Equivalent Ratio for crop *X* as provided by authors	Continuous	—	1557 (50.4%)
	LER_crop_X_calc	Partial Land Equivalent Ratio for crop *X* recalculated from yield data	Continuous	—	2212 (71.6%)
	LER_tot_calc	Total Land Equivalent Ratio recalculated from yield data	Continuous	—	937 (60.7%)

Total number (percentage) of available and missing data for these attributes across all treatments. The full table is available in the data repository.

## Methods

### Literature search

**Fig. 2:**
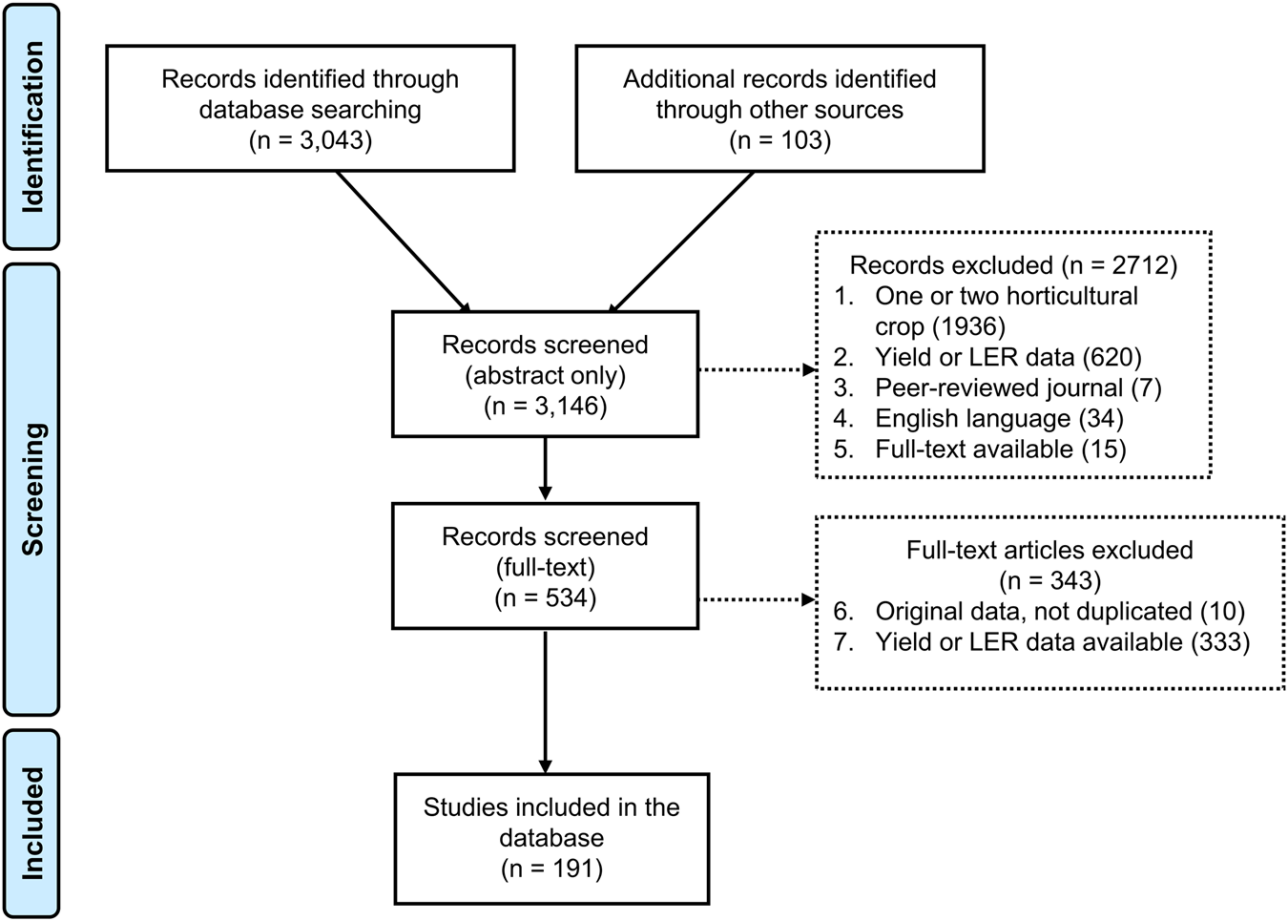
PRISMA diagram of paper selection through the different phases of the systematic literature review. The number of records identified, included and excluded, and the reasons for exclusion are indicated between parentheses.

A systematic literature search was carried out for articles experimenting intercropping that include horticultural crops. Although the term “horticultural” may include ornamental, aromatic or medicinal plants, we limited our scope of investigation to fruits and vegetables crops. The literature search was carried out in October 16, 2023, on papers published up to and including the year 2022, on the Web of Science search engine. The search equation was as follows: TS = ((intercrop* OR inter crop* OR agroforest* OR agro-forest* OR “agr*s*lv*cult*” OR agrihortisilvicult* OR “woody polycultur*” OR “mixed crop*” OR “alley crop*” OR “home garden*” OR “forest garden*” OR “multilayer tree garden*” OR “fruit-vegetable crop*”) AND (fruit* OR orchard* OR vegetable* OR legume* OR “market garden*” OR horticultur*) AND (LER OR “land equivalent ratio” OR yield* OR “agronomic performanc*” OR productivity OR profitability)). The literature search identified 3043 articles as of potential interest. In addition, other articles were identified through others sources (e.g. references cited in the selected articles) and were included if they were relevant to the same criteria. Each article was examined to determine if it met our inclusion criteria. The criteria used to include papers in our corpus were: (1) article title or abstract reporting at least one horticultural crop (fruit or vegetable) and not more than two crops, grown in intercropping and as sole crops (N.B. for crops that can be considered both horticultural and field crops (e.g. maize), we verified that the crop was associated with a horticultural crop, to avoid including field crop studies, a list of unique intercropping systems included in the database is provided in the data repository); (2) article title or abstract reporting at least one experiment conducted with yield and/or LER data collected; (3) article published in a peer-reviewed journal; (4) article written in English and (5) full-text article available in open access or through author’s institutional access. From the 534 full-text articles that met these first criteria, the eligible articles were then screened according to additional criteria: (6) full-text article reporting raw data not duplicated in other articles; (7) full-text article reporting total and/or commercial yield for crops as intercrops and as sole crops, or land equivalent ratios. In addition, the CRAAP^18^ framework (Currency, Relevance, Authority, Accuracy and Purpose) was applied to check the quality of papers to be included in the database. The paper selection process is reported in the PRISMA^19^ diagram (Fig. 2) and the PRISMA checklist was used to report the systematic review. We finally ended up with 191 full-text articles^20–210^ that met all seven criteria, published between 1982 and 2022.

### Data extraction and collection

Data was extracted from tables, graphs or text. Values reported in graphs were digitized manually with the WebPlotDigitizer application (https://automeris.io/WebPlotDigitizer/). When data were not reported for some variables (e.g., Land Equivalent Ratio), we systematically recalculated data from related variables in order to retrieve the missing data. Data are recorded as a CSV-formatted file. This format is widely supported by spreadsheets and enhance data interoperability for scientific applications. The different field site * years * treatment * control combinations are presented in rows, including the row header for the names of variables. All years of data were extracted corresponding to the same control-treatment pairs over different years. Columns represent all variables collected for each treatment. Data collected are grouped into 5 variable groups. Table 1 presents an extract of the variables collected from the articles; the full table is presented in the data repository.

## Data Records

The data are accessible on the Data INRAE repository^211^, available at 10.57745/HV33V1. It includes the following files:

1. “Database.csv” includes the data in “.csv” format.
2. “Database.xlsx” includes the data in Microsoft Excel® format.
3. “Summary of the database.csv” includes a summary of the dataset (meta-data), presenting the name, the definition, the unit and the availability of all extracted variables
4. “List of references.pdf”, presents all the references of the publications included in the dataset
5. “Paut_data_paper_figures_code.Rmd” includes an R script to generate figures.
6. “Classification of F&V.xlsx” includes a fruit and vegetable classification table used in the R script
7. “List of unique intercrops.xlsx” presents a list of the unique intercropping systems included in the database

### General information on the articles

#### ‘Title’, ‘Authors’, ‘DOI’ and ‘year_of_publication’ variables

These variables include basic information allowing to easily retrieve the articles integrated in our database: full title, Authors, DOI (Digital Object Identifier), year of publication.

### Experimental site soil and climate conditions

#### ‘Country’, ‘Latitude’, ‘Longitude’ and ‘Geocoordinates’ variables

These variables present site-related information: country where experiments were carried out, latitude and longitude (in decimal degrees) of experimental sites. The lat-long coordinates were either extracted directly from the paper, or estimated based on site name identified in Google Maps (https://maps.google.com/). The ‘Geocoordinates’ variable reports whether the lat-long coordinates were exact data directly extracted from the paper or estimated through Google Maps.

#### ‘Climate_zone_Koppen_Geiger’, ‘Soil type’, Soil_texture’ and Soil_pH’ variables

These variables include main pedo-climatic conditions. The climate zone ‘*Climate_zone_Koppen_Geiger’* is coded according to Köppen-Geiger climate classification^13^, the soil texture *‘Soil_texture’* is recoded according to the USDA soil texture calculator (https://www.nrcs.usda.gov/resources/education-and-teaching-materials/soil-texture-calculator), soil type *‘Soil_type*’ is recoded according to the World Reference Base for Soil Resources (https://www.fao.org/3/i3794en/I3794en.pdf) or the USDA Soil Taxonomy classification (https://lod.nal.usda.gov/nalt/216302), depending on what the authors refer to, prefixes “WRB” and “USDA” are given respectively. Soil pH *‘Soil_pH’* is reported when available in papers.

### Crop management practices

#### ‘Greenhouse’, ‘Organic_ferti’, ‘Mineral_ferti’, ‘Pesticide_use’ and ‘Irrigation’ variables

These variables present the most commonly reported management practices that may influence intercropping system performances. The variables: greenhouse conditions (‘*Greenhouse’)*, use of organic *(‘Organic_ferti’)* and mineral fertilizers *(‘Mineral_ferti’)*, use of pesticides *(‘Pesticide_use’)*, use of irrigation *(‘Irrigation’)* are all reported as binary variables (Yes/No).

### Intercropping design descriptors

#### ‘Intercropping_design’

**Fig. 3:**
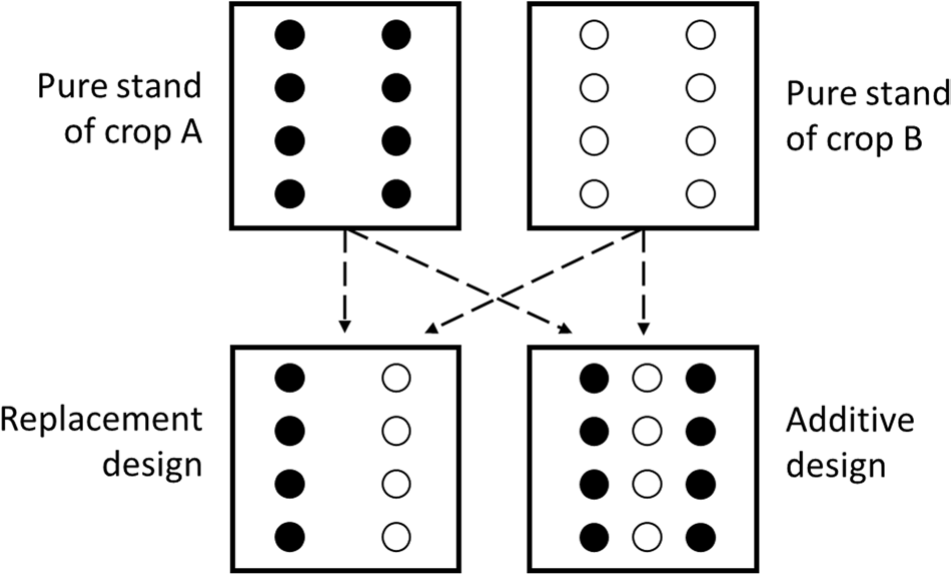
Planting arrangements for pure stand of crop A (●) and crop B (○) for replacement and additive designs (adapted from Snaydon 1991^222^).

This variable presents in which way both species were intercropped (Fig. 3):

I. Replacement (or substitutive) design: In the standard replacement design, intercropping systems are formed by replacing a given number of plants of one component by the same number of the other component. As a result, the density of each component is less in the mixture than in its pure stand, but the total stand density is the same in the mixture as in each pure stand^17^.
II. Additive design: In the standard additive design, intercropping systems are formed by adding plant densities of respective pure stands. As a result, the total density is greater in the intercropping than in the pure stand, but the density of each component is the same in the mixture as in the pure stand.

#### ‘Intercropping_pattern’

**Fig. 4:**
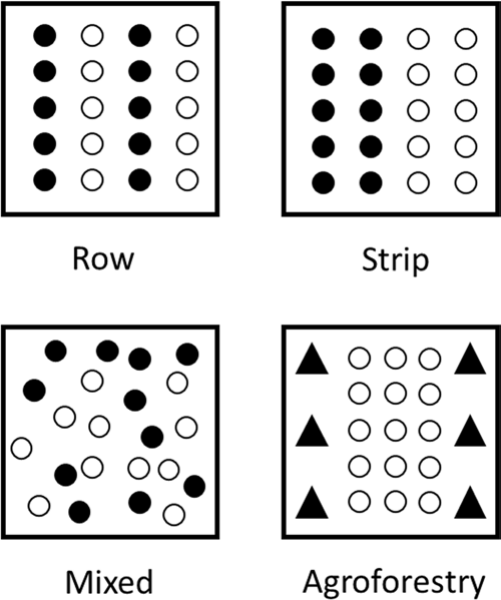
Planting arrangements for pure stand of crop A (●) and crop B (○) for row, strip and mixed design, and for the specific case of agroforestry with trees (▲).

This variable presents in which way both species were spatially sown^212^ (Fig. 4): (i) Row intercropping, where two plant species are cultivated in separate alternate rows; (ii) Strip intercropping, where several rows of a plant species are alternated with one or several rows of another plant species (one strip includes more than one row), (iii) Mixed intercropping, where the component crops are planted simultaneously within the same row or without a distinct row or strip pattern, (iv) Agroforestry. All the agroforestry systems were alley cropping systems^213,214^.

### Crop and yield variables

Since the intercropping experiments include two species, the demonstration is made for one specie (called Crop_1) and is replicable for the second specie (Crop_2).

#### ‘Crop_1_Common_Name’ and ‘Crop_1_Scientific_Name’

These variables give species scientific and common names. The scientific name of each species was related to the common name listed in the United States Department of Agriculture Plants Database (http://plants.usda.gov/java/), to avoid confusion due to the use of different common names for the same species (Table 2).

**Table 2:** Summary table of most common species included in the database.

Common name	Scientific name	Botanical family	Number of experiments	Number of articles
Maize	*Zea mays L*.	Poaceae	624	77
Bean	*Phaseolus L*.	Fabaceae	293	41
Cowpea	*Vigna unguiculata L. Walp*.	Fabaceae	216	34
Tomato	*Solanum lycopersicum L*.	Solanaceae	165	19
Lettuce	*Lactuca sativa L*.	Asteraceae	119	20
Onion	*Allium cepa L*.	Alliaceae	76	18
Pigeon pea	*Cajanus cajan L. Millsp*.	Fabaceae	76	12
Apple	*Malus pumila Mill*.	Rosaceae	74	3
Pepper	*Capsicum annuum L*.	Solanaceae	74	13
Fava bean	*Vicia faba L*.	Fabaceae	71	10
Pea	*Pisum sativum L*.	Fabaceae	61	9
Carrot	*Daucus carota L. var. sativus Hoffm*.	Apiaceae	54	13
Potato	*Solanum tuberosum*	Solanaceae	53	9
Mung bean	*Vigna radiata L*.	Fabaceae	48	13
Peanut	*Arachis hypogaea L*.	Fabaceae	48	12
Cucumber	*Cucumis sativus L*.	Cucurbitaceae	47	6
Pearl millet	*Pennisetum americanum L. Leeke*	Poaceae	45	3
Cassava	*Manihot esculenta Crantz*	Euphorbiaceae	42	6
Beetroot	*Beta vulgaris L*.	Amaranthaceae	41	9
Eggplant	*Solanum melongena L*.	Solanaceae	40	5
Okra	*Abelmoschus esculentus*	Malvaceae	37	6
Banana	*Musa L*.	Musaceae	35	5
Cabbage	*Brassica oleracea L*.	Brassicaceae	33	5
Cluster bean	*Cyamopsis tetragonoloba L. Taubert*	Fabaceae	33	5
Cauliflower	*Brassica oleracea L. var. italica*	Brassicaceae	31	6
Groundnut	*Apios americana Medik*.	Fabaceae	31	8
Millet	*Pennisetum glaucum*	Poaceae	27	2
Wheat	*Triticum aestivum L*.	Poaceae	27	5
Collard	*Brassica oleracea L. var. acephala DC*.	Brassicaceae	26	3
Lablab bean	*Lablab purpureus L*.	Fabaceae	26	7
Radish	*Raphanus sativus L*.	Brassicaceae	24	11
Safed musli	*Chlorophytum borivilianum*	Asparagaceae	24	1
Fenugreek	*Trigonella foenum-graecum L*.	Fabaceae	23	5
Black gram	*Vigna mungo L. Hepper*	Fabaceae	22	7
Garlic	*Allium sativum L*.	Alliaceae	22	10

Data with species with less than 20 entries are not shown.

#### ‘Crop_1_yield_sole’

This variable gives the yield of Crop 1 when grown as sole crop. The yield is the harvestable yield, or when provided the commercial yield; which differs from the harvestable yield by subtracting non-marketable crops. Yield unit is kept as provided in the original paper.

#### ‘Crop_1_yield_intercropped’

This variable gives the yield of Crop 1 when grown in intercropping. The unit is kept as provided in the original paper.

#### ‘Yield_unit’ and ‘Yield_measure’

The variable *Yield_unit* gives the yield unit as provided by authors. Although it is mostly Kg per hectare or ton per hectare, it can sometimes be more specific (*e.g*. ton per feddan) or even not normalized (*e.g*., Kg per plant). The variable ‘*Yield_measure’* indicates what type of yield was recorded (e.g., grain yield, dry weight, marketable yield, etc.).

#### ‘LER_crop_1’, ‘LER_tot’, ‘LER_crop_1_calc’ and ‘LER_tot_calc’

**Fig. 5:**
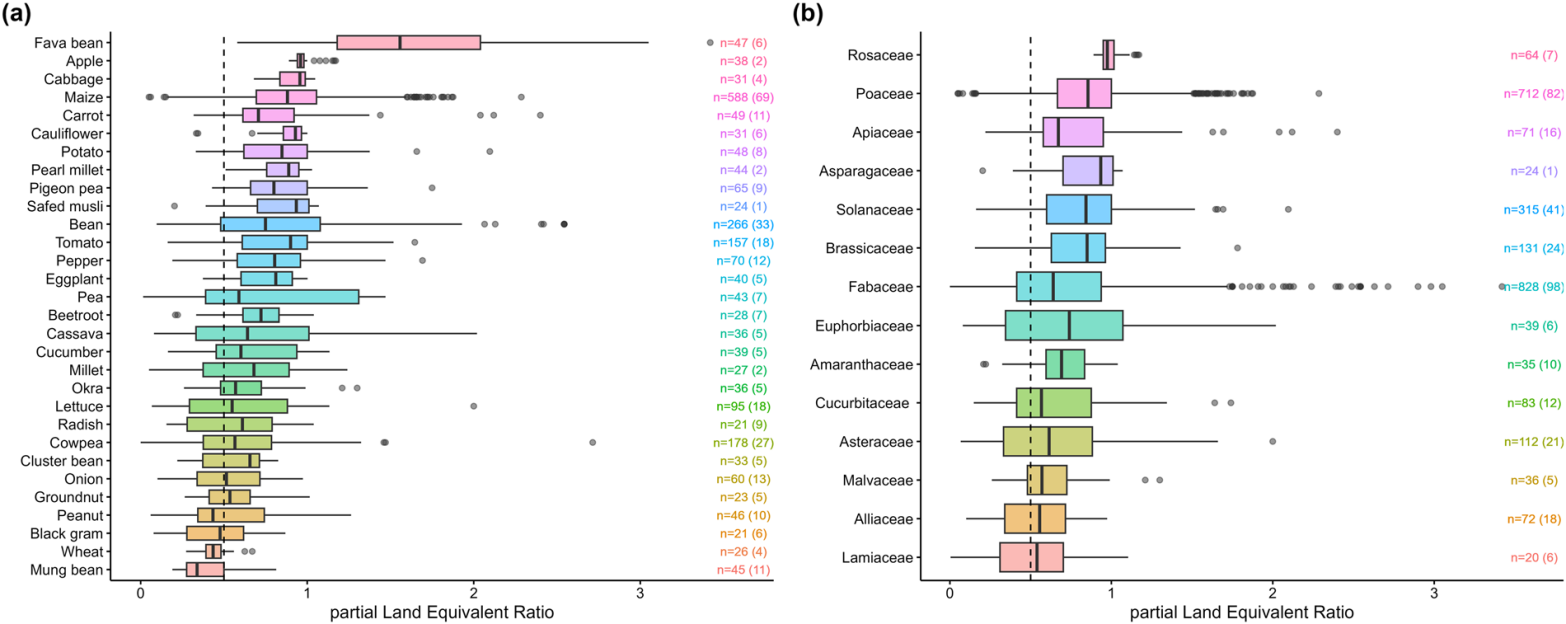
Distribution of partial Land Equivalent Ratios (pLER) for the most represented species in our database (**a**) and for the species grouped by botanical family (**b**). The median is represented by vertical bars inside boxes. Box edges indicate first and third quartile, whiskers indicate minimum and maximum values. The dashed line represents a pLER of 0.5. A pLER Value greater than 0.5 indicates a yield advantage for the intercrop compared to sole crop. The number indicated for each modality is the total number of observations and the number between parentheses is the number of articles. Species and botanical families are ranked in descending order of median values. Modalities with less than 20 occurrences are not plotted.

The variable *‘LER_tot’* is the total Land Equivalent Ratio, which is the sum of partial LERs ‘*LER_crop_1’ and ‘LER_crop_2’*. The partial and total LERs were reported by the raw value provided by the paper, but were also recalculated (‘LER_crop_1_calc’ and ‘LER_tot_calc’) according to Eq. 1). The Land Equivalent Ratio is a widely used indicator to assess intercropping performances^215^ calculated as follows:

1 $$LE{R}_{tot\_calc}=LE{R}_{crop\_1\_calc}+LE{R}_{crop\_2\_calc}=\frac{{Y}_{1}}{{S}_{1}}+\frac{{Y}_{2}}{{S}_{2}}$$

where *LER*_*tot_calc*_ is the calculated total Land Equivalent Ratio, *LER*_*crop n*_ is the partial LER of crop *n*, *Y*_*n*_ is the yield of crop *n* in intercropping and *S*_*n*_ is yield of crop *n* as sole crop. Figure 5 represents the partial Land Equivalent Ratios of the most represented crops grouped by (a) crop and (b) crop botanical family.

## Technical Validation

The database only contains works that have been published in peer-reviewed journals. The same individual thoroughly reviewed each publication to assess its eligibility and the reliability of data. In total, 122 (64% of the total number) papers were checked by at least two different readers to avoid any errors. All co-authors participated in the selection and reading of the papers. A kappa test was performed between the two main contributors to test interrater reliability^216^. From a sample of 101 randomly selected papers, we obtained a kappa score of 0.839, which is considered as strong agreement. For numerical data in the text or in tables, the values provided are directly from the primary data, for figures, WebPlotDigitizer (automeris.io/WebPlotDigitizer/) program was used, allowing for a semi-automatic and more precise extraction of data presented in figures. During data extraction, outliers were routinely and manually examined for possible errors by recalculating the data (Eq. 1). We checked the data’s validity as many times as necessary by going back to the source publications. We deleted studies when the meaning of the data reported in the articles was unclear (e.g., no unit on yield data, no consistency in the names of the different treatments or too approximate description of the protocol that made it impossible to identify the different treatments with certainty). Once the dataset was constructed, we checked the qualitative and quantitative content of all the continuous, categorical and binary variables (Table 1). We checked for variable format, range, factor levels, uniqueness and valid/missing observations information. The check was carried out by two of the co-authors. Finally, variable attributes were checked by a visual assessment of the summary statistics and data distribution for each variable in turn with the *summarytools* R package (v. 0.6.5). A summary table is provided in the data repository.

## Usage Notes

The dataset is based on a collection of experimental data published in 191 articles between 1982 and 2022. It is to our knowledge the first and most exhaustive dataset on intercropping and agroforestry studies exclusively in horticulture. We identify four potential uses of the dataset that could benefit alternatively researchers in agronomy and ecology, agricultural advisors or farmers.

First, the dataset can be analyzed to evaluate the agronomic performance of a wide variety of fruit and vegetable species in intercropping. We can already anticipate that this dataset can be used to identify the best performing species or to better understand the effect of variables of interest: environmental variables (climate, soil), intercropping design (row, strip, replacement, etc.), management practices (fertilization, irrigation, etc.). The data provided could be particularly suitable for a meta-analysis of variance^217,218^, or as input data for intercrop models^219,220^.

**Fig. 6:**
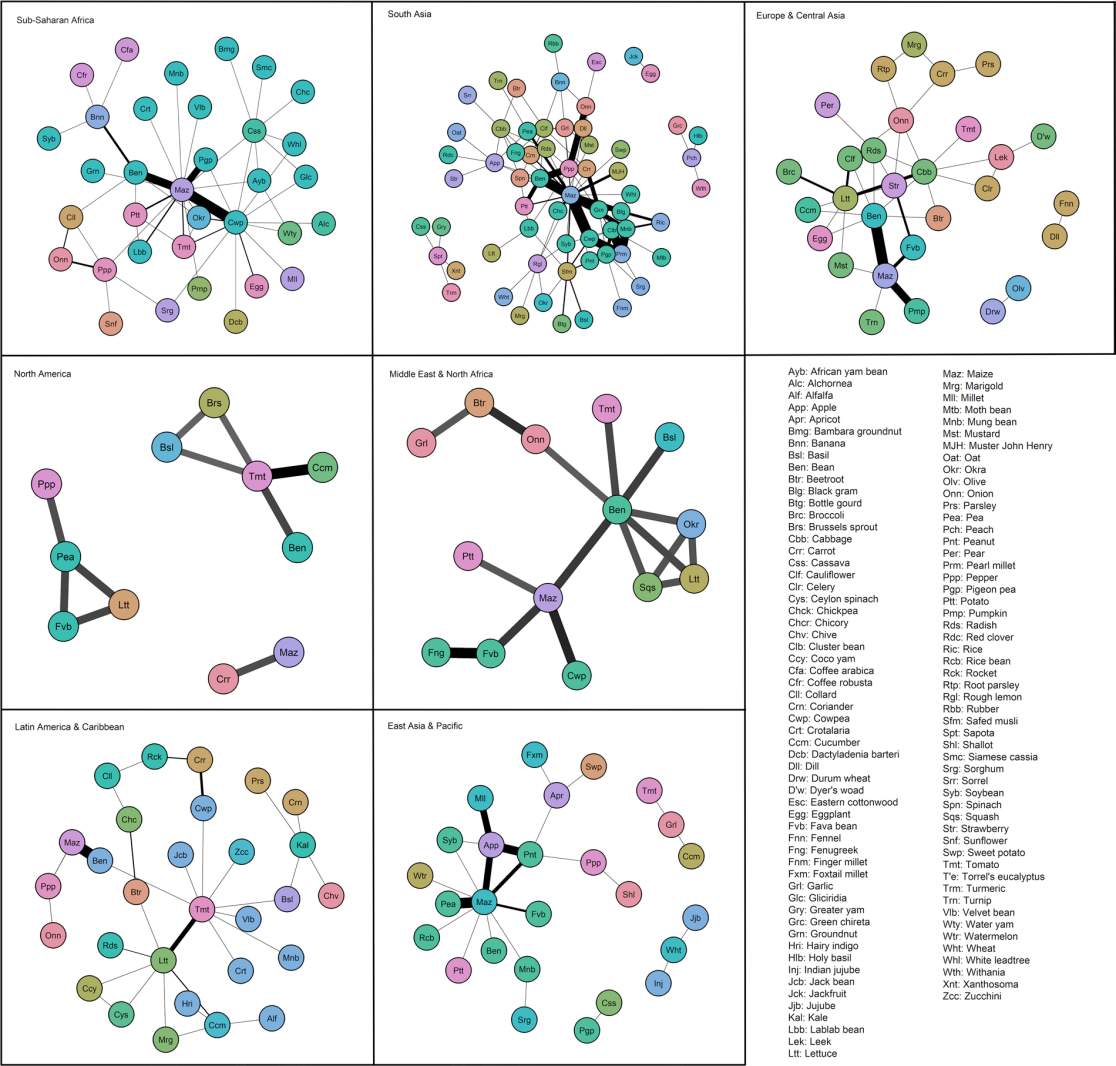
Regional networks of horticultural species included in the database. The regions considered are regions defined in the World Bank Development Indicators. The edges (links) between two species represent a pair of species grown in intercropping experiments. Edges width is proportional to the number of field experiments. Nodes with similar colors are crops from the same botanical families.

Second, a more exploratory approach may be used with our dataset. Figure 6 illustrates the geographical differences in intercropping studies by displaying regional networks (subsets of the dataset) of species pairs. This data may be useful to solve local problems, target the right species under specific climatic and soil conditions, and determine which species allow for accurate comparisons and which have insufficient information. Also, the database can serve as a tool for comparing assessments of species production in contexts with biotic and abiotic yield-limiting stresses in the context of climate change^221^.

Finally, the location of the trials was systematically reported through latitude and longitude coordinates. This makes it simple to connect our database to other large-scale databases, such as soil (*e.g*. data.isric.org/), climate (*e.g*. worldclim.org) or agroecological zone classifications (*e.g*. gaez.fao.org/).

The dataset is freely reusable and easy to update. The data file is structured in such a way that it is straightforward to add new studies from alternative scientific databases, grey literature, or new variables to be investigated to extend the range of possible applications of our dataset.

## Data Availability

Scripts used with R programming language and additional related files are provided at: 10.57745/HV33V1.

## References

[1] Ray DK, Ramankutty N, Mueller ND, West PC, Foley JA (2012). Recent patterns of crop yield growth and stagnation. Nat. Commun..

[2] van Dijk M, Morley T, Rau ML, Saghai Y (2021). A meta-analysis of projected global food demand and population at risk of hunger for the period 2010–2050. Nat. Food.

[3] Mora O (2020). Exploring the future of land use and food security: A new set of global scenarios. PLoS One.

[4] Billen, G., Lassaletta, L. & Garnier, J. A vast range of opportunities for feeding the world in 2050: Trade-off between diet, N contamination and international trade. *Environ. Res. Lett*. **10**, (2015).

[5] Vogel, E. *et al*. The effects of climate extremes on global agricultural yields. *Environ. Res. Lett*. **14**, (2019).

[6] Tilman D, Balzer C, Hill J, Befort BL (2011). Global food demand and the sustainable intensification of agriculture. Proc. Natl. Acad. Sci..

[7] Tibi, A., Martinet, V. & Vialatte, A. *Protect crops by increasing plant diversity in agricultural areas*. *Summary report of the collective scientific assessment*. https://hal.inrae.fr/hal-03852254 10.17180/4F13-NW36 (2022).

[8] Tamburini G (2020). Agricultural diversification promotes multiple ecosystem services without compromising yield. Sci. Adv..

[9] Beillouin, D., Ben-Ari, T. & Makowski, D. Evidence map of crop diversification strategies at the global scale. *Environ. Res. Lett*. **14**, (2019). 10.1016/j.dib.2019.103898 PMC6465772 31016217

[10] Beillouin D, Ben-Ari T, Malézieux E, Seufert V, Makowski D (2021). Positive but variable effects of crop diversification on biodiversity and ecosystem services. *Glob*. Chang. Biol..

[11] Li C (2020). Syndromes of production in intercropping impact yield gains. Nat. Plants.

[12] Li L, Tilman D, Lambers H, Zhang FS (2014). Plant diversity and overyielding: Insights from belowground facilitation of intercropping in agriculture. New Phytol..

[13] Wu J, Liu W, Chen C (2016). Below-ground interspecific competition for water in a rubber agroforestry system may enhance water utilization in plants. Sci. Rep..

[14] Li X-F (2021). Long-term increased grain yield and soil fertility from intercropping. Nat. Sustain..

[15] Khan ZR (1997). Intercropping increases parasitism of pests. Nature.

[16] Ratnadass A, Fernandes P, Avelino J, Habib R (2012). Plant species diversity for sustainable management of crop pests and diseases in agroecosystems: a review. Agron. Sustain. Dev..

[17] Beck HE (2018). Present and future köppen-geiger climate classification maps at 1-km resolution. Sci. Data.

[18] Korber, I. *Evaluating Information – Applying the CRAAP Test*. https://library.csuchico.edu/sites/default/files/craap-test.pdf (2010).

[19] Page, M. J. *et al*. The PRISMA 2020 statement: An updated guideline for reporting systematic reviews. *BMJ* **372**, (2021). 10.1136/bmj.n71 PMC8005924 33782057

[20] Carrubba A, Torre R, Saiano F, Aiello P (2008). Sustainable production of fennel and dill by intercropping. Agron. Sustain. Dev..

[21] Ngwira AR, Aune JB, Mkwinda S (2012). On-farm evaluation of yield and economic benefit of short term maize legume intercropping systems under conservation agriculture in Malawi. F. Crop. Res..

[22] Olasantan FO (1998). Effects of preceding maize (Zea mays) and cowpea (Vigna unguiculata) in sole cropping and intercropping on growth, yield and nitrogen requirement of okra (Abelmoschus esculentus). J. Agric. Sci..

[23] Senaratne R, Liyanage NDL, Ratnasinghe DS (1993). Effect of K on nitrogen fixation of intercrop groundnut and the competition between intercrop groundnut and maize. Fertil. Res..

[24] Srivastava AK, Huchche AD, Ram L, Singh S (2007). Yield prediction in intercropped versus monocropped citrus orchards. Sci. Hortic. (Amsterdam)..

[25] Kermah M (2017). Maize-grain legume intercropping for enhanced resource use efficiency and crop productivity in the Guinea savanna of northern Ghana. F. Crop. Res..

[26] Workayehu T, Wortmann CS (2011). Maize-bean intercrop weed suppression and profitability in Southern Ethiopia. Agron. J..

[27] Yildirim E, Guvenc I (2005). Intercropping based on cauliflower: More productive, profitable and highly sustainable. Eur. J. Agron..

[28] Cecílio Filho AB, Rezende BLA, Barbosa JC, Grangeiro LC (2011). Agronomic efficiency of intercropping tomato and lettuce. Ann. Brazilian Acad. Sci..

[29] Álvarez-Solís JD, Mendoza-Núñez JA, León-Martínez NS, Castellanos-Albores J, Federico A G-M (2016). Effect of bokashi and vermicompost leachate on yield and quality of pepper (Capsicum annuum) and onion (Allium cepa) under monoculture and intercropping cultures. Cienc. e Investig. Agrar..

[30] Syafruddin & Suwardi. Intercropping of maize-mungbean to increase the farmer’s income. *IOP Conf. Ser. Earth Environ. Sci*. **484**, (2020).

[31] Favacho FS, de Lima JSS, Neto FB, da Silva JN, Barros AP (2017). Productive and economic efficiency of carrot intercropped with cowpeavegetable resulting from green manure and different spatial arrangements. Rev. Cienc. Agron..

[32] Fonseca MCM (2016). Lettuce and marigold intercropping: crops productivity and marigold’s flavonoid content. Ciência Rural.

[33] Obiagwu CJ (1995). Estimated yield and nutrient contributions of legume cover crops intercropped with yam, cassava, and maize in the benue river basins of nigeria. J. Plant Nutr..

[34] Singh M, Singh A, Singh S, Tripathi RS, Patra DD (2011). Production potential and economics of safed musli (Chlorophytum borivilianum) under intercropping system. Arch. Agron. Soil Sci..

[35] Hu F (2016). Boosting system productivity through the improved coordination of interspecific competition in maize/pea strip intercropping. F. Crop. Res..

[36] Wangiyana, W., Jaya, I. K. D. & Suheri, H. Application of Mycorrhiza-based biofertilizer to increase yields of several varieties of small chili intercropped with peanut or shallot. *IOP Conf. Ser. Earth Environ. Sci*. **648**, (2021).

[37] Zhang W (2019). Competition for 15N-labeled nitrogen in a jujube tree (Zizyphus jujuba Mill.)/wheat (Triticum aestivum L.) agroforestry system in northwestern China. Agrofor. Syst..

[38] Nissen TM, Midmore DJ, Cabrera ML (1999). Aboveground and belowground competition between intercropped cabbage and young Eucalyptus torelliana. Agrofor. Syst..

[39] Bhardwaj A, Kaur N, Dhat AS, Gill RIS (2021). Optimization of onion planting time and variety under Populus deltoides-based agroforestry system in North-Western India. Agrofor. Syst..

[40] Bantie, Y. B. Determination of Effective Spatial Arrangement for Intercropping of Maize (Zea mays L.) and Potato (Solanum tuberosum L.) Using Competition Indices Ethiopia. *J. Hortic*. **02**, (2015).

[41] Panozzo A, Bernazeau B, Desclaux D (2020). Durum wheat in organic olive orchard: good deal for the farmers?. Agrofor. Syst..

[42] Kabura BH, Musa B, Odo PE (2008). Evaluation of the yield components and yield of onion (Allium cepa L.)-pepper (Capsicum annuum L.) intercrop in the Sudan Savanna. J. Agron..

[43] Choudhary VK, Dixit A, Kumar PS, Chauhan BS (2014). Productivity, weed dynamics, nutrient mining, and monetary advantage of maize-legume intercropping in the Eastern Himalayan Region of India. Plant Prod. Sci..

[44] Schultz B (1987). Effects of Planting Densities, Irrigation, and Hornworm Larvae on Yields in Experimental Intercrops of Tomatoes and Cucumbers. J. Am. Soc. Hortic. Sci..

[45] Rezende GDSP, Ramalho MAP (1994). Competitive Ability of Maize and Common Bean (Phaseolus Vulgaris) Cultivars Intercropped in Different Environments. J. Agric. Sci..

[46] Ribas, R. G. T., *et al* Land equivalent ratio in the intercropping of cucumber with lettuce as a function of cucumber population density. *Agric*. **10**, (2020).

[47] Adeniyi O (2011). Economic aspects of intercropping systems of vegetables (okra, tomato and cowpea). Afr. J. Agric. Res.

[48] Cecílio Filho AB, Medelo MJY, DE PONTES SC, Nascimento CS (2021). Chicory and arugula in intercropping with collard greens. Rev. Caatinga.

[49] de Lima JSSS (2014). Agroeconomic evaluation of intercropping rocket and carrot by uni- and multivariate analyses in a semi-arid region of Brazil. Ecol. Indic..

[50] Dua VK, Kumar S, Jatav MK (2017). Effect of nitrogen application to intercrops on yield, competition, nutrient use efficiency and economics in potato (Solanum Tuberosum L.) plus French bean (Phaseolus Vulgaris L.) system in north-western hills of India. Legum. Res..

[51] Rezaei-Chiyaneh E (2021). Vermicompost Application in Different Intercropping Patterns Improves the Mineral Nutrient Uptake and Essential Oil Compositions of Sweet Basil (Ocimum basilicum L.). J. SOIL Sci. PLANT Nutr..

[52] Guldan SJ, Martin CA, Falk CL (1998). Interseeding Snap Pea into Stands of Chile Pepper Reduces Yield of Pea More Than That of Chile. HortScience.

[53] Cenpukdee U, Fukai S (1992). Cassava/legume intercropping with contrasting cassava cultivars. 1. Competition between component crops under three intercropping conditions. F. Crop. Res..

[54] Rapholo, E. *et al*. Maize-lablab intercropping is promising in supporting the sustainable intensification of smallholder cropping systems under high climate risk in southern Africa. *Exp. Agric*. 1–14 (2019) 10.1017/S0014479719000206.

[55] Banik P, Sharma RC (2009). Yield and resource utilization efficiency in baby cornlegume-intercropping system in the eastern plateau of India. J. Sustain. Agric..

[56] Liu T, Cheng Z, Meng H, Ahmad I, Zhao H (2014). Growth, yield and quality of spring tomato and physicochemical properties of medium in a tomato/garlic intercropping system under plastic tunnel organic medium cultivation. Sci. Hortic. (Amsterdam)..

[57] Rodrigo VHL, Stirling CM, Teklehaimanot Z, Nugawela A (1997). The effect of planting density on growth and development of component crops in rubber/banana intercropping systems. F. Crop. Res..

[58] Bhat R, Wani WMM, Sharma MKK, Ashraf N (2014). Studies on Intercropping with Leguminous and Non-leguminous Crops on Yield, Leaf Nutrient Status and Relative Economic Yield of Apple cv. Red Delicious. Int. J. Hortic..

[59] Gitari HI (2020). Revisiting intercropping indices with respect to potato-legume intercropping systems. F. Crop. Res..

[60] Midega CAO (2014). Cumulative effects and economic benefits of intercropping maize with food legumes on Striga hermonthica infestation. F. Crop. Res..

[61] Singh A, Weisser WW, Hanna R, Houmgny R, Zytynska SE (2017). Reduce pests, enhance production: benefits of intercropping at high densities for okra farmers in Cameroon. Pest Manag. Sci..

[62] Morgado LB, Willey RW (2003). Effects of plant population and nitrogen fertilizer on yield and efficiency of maize-bean intercropping. Pesqui. Agropecu. Bras..

[63] Motagally FMFA, Metwally AK (2014). Maximizing Productivity by Intercropping Onion on Sugar Beet. Asian J. Crop Sci..

[64] Sharma RC, Banik P (2015). Baby Corn-Legumes Intercropping Systems: I. Yields, Resource Utilization Efficiency, and Soil Health. Agroecol. Sustain. Food Syst..

[65] Cecílio Filho AB, Rezende BLA, Costa CC (2010). Economic analysis of the intercropping of lettuce and tomato in different seasons under protected cultivation. Hortic. Bras..

[66] Karlidag H, Yildirim E (2009). Strawberry Intercropping with Vegetables for Proper Utilization of Space and Resources. J. Sustain. Agric..

[67] Kabiraj J, Das R, Das SP, Mandal AR (2017). A Study on Cauliflower (Brassica oleracea var. botrytis) Based Intercropping System. Int. J. Curr. Microbiol. Appl. Sci..

[68] Chabi-Olaye A, Nolte C, Schulthess F, Borgemeister C (2006). Relationships of soil fertility and stem borers damage to yield in maize-based cropping system in Cameroon. Ann. la Soc. Entomol. Fr..

[69] Schwerdtner U, Spohn M (2021). Interspecific root interactions increase maize yields in intercropping with different companion crops. J. PLANT Nutr. SOIL Sci..

[70] Mandal BK, Dhara MC, Mandal BB, Das SK, Nandy R (1990). Rice, Mungbean, Soybean, Peanut, Ricebean, and Blackgram Yields under Different Intercropping Systems. Agron. J..

[71] Mondal MRI, Begum F, Alam MM (2012). Study on Intercropping Carrot with Groundnut Under Different Row Arrangements. Bangladesh J. Agric. Res..

[72] Hendges ARADA, Guimarães MDA, Dovale JC, Neto BPL (2019). Agronomic performance and biological efficiency of kale intercropped with spice species. Rev. Caatinga.

[73] Nyawade, S. O. *et al*. Yield and evapotranspiration characteristics of potato-legume intercropping simulated using a dual coefficient approach in a tropical highland. *F. Crop. Res*. **274**, (2021).

[74] Anjum MA, Qasim SA, Ahmad S, Hussain S (2015). Assessment of advantages of pea and non-legume winter vegetable intercropping systems through competition and economic indices. Exp. Agric..

[75] Ribeiro GM (2017). Agro-economic efficiency of the intercropping of carrot X cowpea-vegetable under different spatial arrangements and population densities. Rev. Caatinga.

[76] Tripathi AK, Singh AK (2014). Productivity, economic viability and energy efficiency of intercropping winter maize (Zea mays) and rajmash bean (Phaseolus vulgaris) in potato (Solanum tuberosum) with border ridge technique. Curr. Adv. Agric. Sci..

[77] Hamdani JS, Suradinata YR (2015). Effects of Row Intercropping System of Corn and Potato and Row Spacing of Corn on the Growth and Yields of Atlantic Potato Cultivar Planted in Medium Altitude. Asian J. Agric. Res..

[78] Islam MN, Akhteruzzaman M, Alom MS, Salim M (2014). Hybrid maize and sweet potato intercropping: a technology to increase productivity and profitability for poor hill farmers in Bangladesh. SAARC J. Agric..

[79] Rusinamhodzi L, Makoko B, Sariah J (2017). Ratooning pigeonpea in maize-pigeonpea intercropping: Productivity and seed cost reduction in eastern Tanzania. F. Crop. Res..

[80] Liu L (2017). Biochar amendments increase the yield advantage of legume-based intercropping systems over monoculture. Agric. Ecosyst. Environ..

[81] Xiao X, Cheng Z, Meng H, Khan MA, Li H (2012). Intercropping with garlic alleviated continuous cropping obstacle of cucumber in plastic tunnel. Acta Agric. Scand. Sect. B - Soil Plant Sci..

[82] Abbes Z, Trabelsi I, Kharrat M, Amri M (2019). Intercropping with fenugreek (Trigonella foenum-graecum) enhanced seed yield and reduced Orobanche foetida infestation in faba bean (Vicia faba). Biol. Agric. Hortic..

[83] Härdter R, Horst WJ (1991). Nitrogen and phosphorus use in maize sole cropping and maize/cowpea mixed cropping systems on an Alfisol in the northern Guinea Savanna of Ghana. Biol. Fertil. Soils.

[84] Yildirim E, Guvenc I (2004). Intercropping in cucumber (Cucumis sativus) under greenhouse conditions. Indian J. Agric. Sci..

[85] Salgado GC (2020). Nitrogen transfer from green manure to organic cherry tomato in a greenhouse intercropping system. J. Plant Nutr..

[86] Huang C (2018). Optimised sowing date enhances crop resilience towards size-asymmetric competition and reduces the yield difference between intercropped and sole maize. F. Crop. Res..

[87] Sarker PK, Rahman MM, Das BC (2007). Effect of Intercropping with Mustard with Onion and Garlic on Aphid Population and Yield. J. Bio-Science.

[88] Thierfelder C, Cheesman S, Rusinamhodzi L (2012). A comparative analysis of conservation agriculture systems: Benefits and challenges of rotations and intercropping in Zimbabwe. F. Crop. Res..

[89] Kidane BZ, Hailu MH, Haile HT (2017). Maize and potato intercropping: A technology to increase productivity and profitability in tigray. Open Agric..

[90] Arshad M (2013). Yield Comparison of Nonstructural Carbohydrates in Sweet Sorghum and Legume-Based Cropping Systems. Commun. Soil Sci. Plant Anal..

[91] Rezaei-Chianeh E (2011). Intercropping of maize (Zea mays L.) and faba bean (Vicia faba L.) at different plant population densities. African J. Agric. Res..

[92] Al-Dalain SA (2009). Effect of Intercropping of Zea Maize with Potato Solanum tuberosum, L. on Potato Growth and on the Productivity and Land Equivalent Ratio of Potato and Zea Maize. Agric. J..

[93] Latati M (2016). The intercropping common bean with maize improves the rhizobial efficiency, resource use and grain yield under low phosphorus availability. Eur. J. Agron..

[94] Yap, V. Y., Xaphokhame, P., de Neergaard, A. & Bruun, T. B. Barriers to Agro-Ecological Intensification of Smallholder Upland Farming Systems in Lao PDR. *AGRONOMY-BASEL* **9**, (2019).

[95] Saidi, M. & Itulya, F. M. Effect of Intercropping Collard with Beans or Onions on Aphid Populations and Yields of Collard Under High Altitude Conditions in Kenya. *Tanzania**J. Agric. Sci*. 57–66 (2008).

[96] Chowdhury MK, Rosario EL (1992). Utilization efficiency of applied nitrogen as related to yield advantage in maize/mungbean intercropping. F. Crop. Res..

[97] Sekamatte BM, Ogenga-Latigo M, Russell-Smith A (2003). Effects of maize-legume intercrops on termite damage to maize, activity of predatory ants and maize yields in Uganda. Crop Prot..

[98] Santillano-Cázares, J. *et al*. Assessment of intercropping and plastic mulch as tools to manage heat stress, productivity and quality of jalapeño pepper. *Agronomy* **8**, (2018).

[99] Zougmore R, Kambou FN, Ouattara K, Guillobez S (2000). Sorghum-cowpea intercropping: An effective technique against runoff and soil erosion in the sahel (saria, burkina faso). Arid Soil Res. Rehabil..

[100] Ahmed M (2016). Hybrid Maize and Chilli Intercropping in the Hilly Areas of Bandarban. Bangladesh Agron. J..

[101] Momirović N (2015). Productivity of intercropping maize (Zea mays L.) and pumpkins (Cucurbita maxima Duch.) under conventional vs. conservation farming system. Turkish J. F. Crop..

[102] Zhang W (2017). Competitive interaction in a jujube tree/wheat agroforestry system in northwest China’s Xinjiang Province. Agrofor. Syst..

[103] Coutinho PWR (2017). Establishment of intercropping of beet and chicory depending on soil management. Rev. Cienc. Agron..

[104] Sarkar RK, Shit D (1993). Effect of Intercropping Cereal, Pulse and Oilseed Crops in Redgram on Yield, Competition and Advantage. J. Agron. Crop Sci..

[105] Newman SM (1986). A Pear and Vegetable Interculture System: Land Equivalent Ratio, Light Use Efficiency and Productivity. Exp. Agric..

[106] Legodi KD, Ogola JBO (2020). Cassava-legume intercrop: I. Effects of relative planting dates of legumes on cassava productivity. Acta Agric. Scand. Sect. B Soil Plant Sci..

[107] Adhikary S, Bagchi DK, Ghosal P, Banerjee RN, Chatterjee BN (1991). Studies on Maize-Legume Intercropping and their Residual Effects on Soil Fertility Status and Succeeding Crop in Upland Situation. J. Agron. Crop Sci..

[108] Zhang W (2019). Different tree age affects light competition and yield in wheat grown as a companion crop in jujube-wheat agroforestry. Agrofor. Syst..

[109] Begum SA, Zaman MS, Khan A (2015). Intercropping of root crops with chilli in charlands of Mymensingh. Progress. Agric..

[110] Chaudhari DT, Vekariye PD, Vora VD, Talpada MM, Sutaria GS (2017). Enhancing productivity of groundnut based intercropping systems under rainfed conditions of Gujarat. Legum. Res..

[111] Hu F (2020). Optimizing the split of N fertilizer application over time increases grain yield of maize-pea intercropping in arid areas. Eur. J. Agron..

[112] Kang BT, Caveness FE, Tian G, Kolawole GO (1999). Longterm alley cropping with four hedgerow species on an Alfisol in southwestern Nigeria - Effect on crop performance, soil chemical properties and nematode population. Nutr. Cycl. Agroecosystems.

[113] Ronner E, Thuijsman E, Ebanyat P, Descheemaeker K, Giller KE (2021). Intercropping of climbing bean (Phaseolus vulgaris, L.) and East African highland banana (Musa spp.) in the Ugandan highlands. Exp. Agric..

[114] Reddy KC, Visser P, Buckner P (1992). Pearl millet and cowpea yields in sole and intercrop systems, and their after-effects on soil and crop productivity. F. Crop. Res..

[115] Silva ÍN (2018). Green manure and spatial arrangement in the sustainability improvement of lettuce-beet intercrops. Rev. Bras. Eng. Agric. e Ambient..

[116] Tripathi P, Shah S, Kashyap SD, Tripathi A (2018). Fruit yield and quality characteristics of high density Prunus persica (L.) Batsch plantation intercropped with medicinal and aromatic plants in the Indian Western Himalayas. Agrofor. Syst..

[117] Miah MG (2018). Transformation of jackfruit (Artocarpus heterophyllus Lam.) orchard into multistory agroforestry increases system productivity. Agrofor. Syst..

[118] Li YY (2009). Intercropping alleviates the inhibitory effect of N fertilization on nodulation and symbiotic N2 fixation of faba bean. Plant Soil.

[119] Ocimati W (2019). Banana leaf pruning to facilitate annual legume intercropping as an intensification strategy in the East African highlands. Eur. J. Agron..

[120] Bavec M, Žuljan M, Robačer M, Bavec F (2012). White cabbage productivity in intercropping production systems. Acta Hortic..

[121] Jørgensen V, Møller E (2000). Intercropping of different secondary crops in maize. Acta Agric. Scand. - Sect. B Soil Plant Sci..

[122] Njira KOW, Semu E, Mrema JP, Nalivata PC (2021). Productivity of pigeon pea, cowpea and maize under sole cropping, legume–legume and legume–cereal intercrops on Alfisols in Central Malawi. Agrofor. Syst..

[123] Tembakazi Silwana T, Lucas EO (2002). The effect of planting combinations and weeding on the growth and yield of component crops of maize/bean and maize/pumpkin intercrops. J. Agric. Sci..

[124] Leishangthem K, Patel BN, Prajapati DD, Pandya HV (2016). Intercropping of Tuber Crops in Young Orchard of Sapota Cv. Kalipatti. J. Tree Sci..

[125] Vilhekar SC, Badole KK, Ghanbahadur MR (2014). Performance and economics of sweet corn as influenced by leafy vegetables intercropping system under rainfed condition. Adv. Res. J. Crop Improv..

[126] Li L (2007). Diversity enhances agricultural productivity via rhizosphere phosphorus facilitation on phosphorus-deficient soils. Proc. Natl. Acad. Sci. USA.

[127] Walia, S. & Kumar, R. Elucidating the yield and quality response of Tagetes minuta L. intercropped with Zea mays L. under different spacing in the western Himalayas. *Ind. Crops Prod*. **171**, (2021).

[128] Baumann DT, Bastiaans L, Kropff MJ (2001). Competition and crop performance in a leek-celery intercropping system. Crop Sci..

[129] Costa APDA (2017). Intercropping of carrot x cowpea-vegetables: evaluation of cultivar combinations fertilized with roostertree. Rev. Caatinga.

[130] Bomford MK (2009). Do Tomatoes Love Basil but Hate Brussels Sprouts? Competition and Land-Use Efficiency of Popularly Recommended and Discouraged Crop Mixtures in Biointensive Agriculture Systems. J. Sustain. Agric..

[131] Sharaiha R, Gliessman S (1992). The Effects of Crop Combination and Row Arrangement in the lntercropping of Lettuce, Favabean and Pea on Weed Biomass and Diversity and on Crop Yields. Biol. Agric. Hortic..

[132] Latati M (2014). The intercropping cowpea-maize improves soil phosphorus availability and maize yields in an alkaline soil. Plant Soil.

[133] Abdel-Wahab SI, Abdel-Wahab EI (2021). Cropping systems of fenugreek with faba bean to reduce broomrape infestation. Legum. Res..

[134] Telles CC, Junqueira AMR, Fukushi YK (2020). de M. Technical feasibility of conventional and nonconventional vegetables’ intercropping under organic fertilization. Biosci. J..

[135] Bai W (2016). Mixing trees and crops increases land and water use efficiencies in a semi-arid area. Agric. Water Manag..

[136] Rajput RL, Rawat GS (2019). Yield and yield attributes as influenced by nutrient management practices in pigeonpea and clusterbean intercropping system. Legum. Res..

[137] Haque MEE, Rahman MAA, Rahman MAA, Roy AKK, Sikdar B (2009). Lablab Bean Based Intercropping System in Northwest Region of Bangladesh. Pakistan J. Biol. Sci..

[138] Mulugeta T (2019). Phosphite alters the behavioral response of potato tuber moth (Phthorimaea operculella) to field-grown potato. Pest Manag. Sci..

[139] Kushwah AS, Rawat GS, Gupta S, Patil D, Prajapati N (2017). Production and profitability assessment of clusterbean [Cyamopsis tetragonoloba (L.) taub.] based intercropping systems under different row arrangement. Legum. Res..

[140] Ogbuehi CRA, Orzolek MD (1987). Intercropping carrot and sweetcorn in a multiple cropping system. Sci. Hortic. (Amsterdam)..

[141] Waweru BW, Rukundo P, Kilalo DC, Miano DW, Kimenju JW (2021). Effect of border crops and intercropping on aphid infestation and the associated viral diseases in hot pepper (Capsicum sp.). Crop Prot..

[142] van Asten PJA, Wairegi LWI, Mukasa D, Uringi NO (2011). Agronomic and economic benefits of coffee–banana intercropping in Uganda’s smallholder farming systems. Agric. Syst..

[143] Tarafder JH, Rahman MS, Hossain AMM, Syeda JA, Rahman MM (2003). Economic Returns and Yield of Chilli as Intercropped with Varying Onion Population. Pakistan J. Biol. Sci..

[144] Bezerra Neto F (2019). Productive viability and profitability of carrot-cowpea intercropping using different amounts of calotropis procera. Rev. CAATINGA.

[145] Grewal SS, Mittal SP, Dyal S, Agnihotri Y (1992). Agroforestry systems for soil and water conservation and sustainable production from foothill areas of north India. Agrofor. Syst..

[146] Osonubi O, Atayese MO, Mulongoy K (1995). The effect of vesicular-arbuscular mycorrhizal inoculation on nutrient uptake and yield of alley-cropped cassava in a degraded Alfisol of southwestern Nigeria. Biol. Fertil. Soils.

[147] Rajput RL, Kushwaha BB (2021). Yield Analysis of Chickpea (Cicer arietinum) with Mustard (Brassaca juncea) Intercropping System as Influenced by Weed Management Practices. Legum. Res..

[148] Kour M, Thakur NP, Kumar P, Charak AS (2016). Productivity and profitability of maize (Zea mays) as influenced by intercropping of rajmash (Phaseolus vulgaris) and nutrient management techniques under sub-alpine conditions of Jammu, India. Legum. Res..

[149] Polthanee A, Trelo-Ges V (2003). Growth, yield and land use efficiency of corn and legumes grown under intercropping systems. Plant Prod. Sci..

[150] Temesgen A, Fukai S, Rodriguez D (2015). As the level of crop productivity increases: Is there a role for intercropping in smallholder agriculture. F. Crop. Res..

[151] Powers LE, McSorley R, Dunn RA, Montes A (1994). The agroecology of a cucurbit-based intercropping system in the Yeguare Valley of Honduras. Agric. Ecosyst. Environ..

[152] Ali Qasim S (2013). Effect of pea intercropping on biological efficiencies and economics of some non-legume winter vegetables. Pakistan J. Agric. Sci..

[153] El-Gaid, M. A. A. A., Al-Dokeshy, M. H. H. & Nassef, D. M. T. T. Effects of Intercropping System of Tomato and Common Bean on Growth, Yield Components and Land Equivalent Ratio in New Valley Governorate. *Asian J. Crop Sci*. 1–8, 10.3923/ajcs.2014 (2014).

[154] Gawade MH, Path JD (2004). Studies on Effect of Intercrops on Yield and Monetary Returns of Cabbage. Agric. Sci. Dig..

[155] Guvenc I, Yildirim E (2006). Increasing Productivity with Intercropping Systems. in Cabbage Production. J. Sustain. Agric..

[156] Olasantan FO (1991). Response of tomato and okra to nitrogen fertilizer in sole cropping and intercropping with cowpea. J. Hortic. Sci..

[157] Sridhar HS, Salakinkop SR (2021). Competitive functions, pest dynamics and bio-economic analysis in traditional maize and legumes intercropping systems under rainfed situation of South India. INDIAN J. Tradit. Knowl..

[158] Varghese L (2000). Indicators of production sustainability in intercropped vegetable farming on montmorillonitic soils in India. J. Sustain. Agric..

[159] Morgado LB, Willey RW (2008). Optimum plant population for maize-bean intercropping system in the Brazilian semi-arid region. Sci. Agric..

[160] Ofori K, Gamedoagbao DK (2005). Yield of scarlet eggplant (Solanum aethiopicum L.) as influenced by planting date of companion cowpea. Sci. Hortic. (Amsterdam)..

[161] Xu H, Bi H, Gao L, Yun L (2019). Alley cropping increases land use efficiency and economic profitability across the combination cultivation period. Agronomy.

[162] Reynafarje X, Siura S, Pérez K (2016). Mixed cropping of vegetables to improve organic tomato (Solanum lycopersicum L.) production in small farmer systems. Acta Hortic..

[163] Brintha I, Seran TH (2012). Effect of intercropping chili (Capsicum annuum L.) with onion (Allium cepa L.) in sandy regosol. *Bangladesh*. J. Agric. Res..

[164] Gao L (2013). Intercropping Competition between Apple Trees and Crops in Agroforestry Systems on the Loess Plateau of China. PLoS One.

[165] Choudhary VK, Dixit A, Chauhan BS (2016). Resource-use maximisation through legume intercropping with maize in the eastern Himalayan region of India. Crop Pasture Sci..

[166] Stavridou E, Kristensen HL, Krumbein A, Schreiner M, Thorup-Kristensen K (2012). Effect of differential N and S competition in inter- and sole cropping of brassica species and lettuce on glucosinolate concentration. J. Agric. Food Chem..

[167] Sharma OP, Gupta AK (2001). Comparing the feasibilities of pearlmillet-based intercropping systems supplied with varying levels of nitrogen and phosphorus. J. Agron. Crop Sci..

[168] Xie Y, Kristensen HL (2017). Intercropping leek (Allium porrum L.) with dyer’s woad (Isatis tinctoria L.) increases rooted zone and agro-ecosystem retention of nitrogen. Eur. J. Agron..

[169] Karlidag H, Yildirim E (2007). The Effects of Nitrogen Fertilization on Intercropped Strawberry and Broad Bean. J. Sustain. Agric..

[170] Kimaro AA, Timmer VR, Chamshama SAO, Ngaga YN, Kimaro DA (2009). Competition between maize and pigeonpea in semi-arid Tanzania: Effect on yields and nutrition of crops. Agric. Ecosyst. Environ..

[171] Çiftçi V, Togay N, Togay Y, Dogan Y (2006). The Effects of Intercropping Sowing Systems with Dry Bean and Maize on Yield and Some Yield Components. J. Agron..

[172] Demir H, Polat E (2011). Effects of broccoli-crispy salad intercropping on yield and quality under greenhouse conditions. African J. Agric. Res..

[173] Rao MR, Singh M (1990). Productivity and risk evaluation in constrasting intercropping systems. F. Crop. Res..

[174] Cecílio Filho AB, Rezende BLA, Canato GHD (2007). Lettuce and radish productivity in intercropping systems as influenced by starting time and row spacings. Hortic. Bras..

[175] Gao H (2020). Yield and nitrogen uptake of sole and intercropped maize and peanut in response to N fertilizer input. Food Energy Secur..

[176] Roltsch WJ, Gage SH (1990). Influence of Bean–Tomato Intercropping on Population Dynamics of the Potato Leafhopper (Homoptera: Cicadellidae). Environ. Entomol..

[177] Schultz B, Phillips C, Rosset P, Vandermeer J (1982). An experiment in intercropping cucumbers and tomatoes in Southern Michigan, USA. Sci. Hortic. (Amsterdam)..

[178] Badawy SAE, Shalaby GA (2015). Effect of intercropping of sugar beet with onion and garlic on insect infestation, sugar beet yield and economics. J. Plant Prod..

[179] Ugrinovic M (2014). Intercropped red beet and radish with green bean affected microbial communities and nodulation by indigenous rhizobia. Agric. Food Sci..

[180] Ziaie-Juybari H, Pirdashti H, Abo-Elyousr KAM, Mottaghian A (2021). Abiotic benefits of intercropping legumes and maize to reduce pests. Arch. Phytopathol. Plant Prot..

[181] Nwofia GE, Jiwuba F, Okpara DA, Mbah EU (2017). Yield and Productivity of Eggplant Genotypes Intercropped with Vegetable Cowpea in the Humid Tropics. Int. J. Veg. Sci..

[182] Choudhary SK, Singh RN, Singh RK, Upadhyay PK (2014). Yield and nutrient uptake of winter maize (Zea mays) with vegetable intercropping. Curr. Adv. Agric. Sci..

[183] De Costa W, Perera M (1998). Effects of bean population and row arrangement on the productivity of chilli/dwarf bean (Capsicum annuum/Phaseolus vulgaris L.) intercropping in Sri Lanka. J. Agron. Crop Sci..

[184] Ranjan R (2021). Evaluation of maize-based intercropping on runoff, soil loss, and yield in foothills of the Indian sub-Himalayas. Exp. Agric..

[185] Biswas, B. *et al*. Agroforestry offers multiple ecosystem services in degraded lateritic soils. *J. Clean. Prod*. **365**, (2022).

[186] Cecílio Filho AB (2022). Intercropping of Eggplant and Tomato As Function of Times of Transplant and Cropping Season. Rev. Caatinga.

[187] Chaves, A. P. *et al*. Bio-agroeconomic returns in beet-cowpea intercropping by optimization of population densities and spatial arrangements. *Acta Sci. Agron*. **44**, (2022).

[188] Cuartero, J. *et al*. A first-year melon/cowpea intercropping system improves soil nutrients and changes the soil microbial community. *Agric. Ecosyst. Environ*. **328**, (2022).

[189] Jiang Y (2022). Evaluation of maize/peanut intercropping effects on microbial assembly, root exudates and peanut nitrogen uptake. Plant Physiol. Biochem..

[190] Ndengu, G. *et al*. Effect of combining organic manure and inorganic fertilisers on maize-bush bean intercropping. *Exp. Agric*. **58**, (2022).

[191] Sheha AM, El-Mehy AA, Mohamed AS, Saleh SA (2022). Different wheat intercropping systems with tomato to alleviate chilling stress, increase yield and profitability. Ann. Agric. Sci..

[192] Wang, X., Cao, B., Zou, J., Xu, A. & Feng, X. Intercropping Gramineae Herbage in Semiarid Jujube Cultivar ‘LingwuChangzao’ (Ziziphus jujuba Mill. cv. LingwuChangzao) Orchard Improves Productivity, Plant Nutritional Quality, and Soil Quality. *Horticulturae* **8**, (2022).

[193] Raffa, D. W., Migliore, M., Campanelli, G., Leteo, F. & Trinchera, A. Effects of Faba Bean Strip Cropping in an Outdoor Organic Tomato System on Soil Nutrient Availability, Production, and N Budget under Different Fertilizations. *Agronomy* **12**, (2022).

[194] Yang, Z. *et al*. Intercropping regulation of soil phosphorus composition and microbially-driven dynamics facilitates maize phosphorus uptake and productivity improvement. *F. Crop. Res*. **287** (2022).

[195] Yimer, T., Abera, G., Beyene, S. & Rasche, F. Optimizing maize-bean cropping systems for sustainable intensification in southern Ethiopia. *Agron. J*. **114**, (2022).

[196] Abou-Hussein SD, Salman SR, Abdel-Mawgoud AMR, Ghoname AA (2005). Productivity, Quality and Profit of Sole or Intercropped Green Bean (Phaseolus vulgaris L.) Crop. J. Agron..

[197] Amin MHA, Sumi S, Jutidamrongphan W, Techato KA (2021). Economic potentiality of Colocasia esculenta L. under multipurpose treebased agroforestry systems. Asian J. Agric. Biol..

[198] Hugar HY, Palled YB (2008). Studies on Maize-Vegetable Intercropping Systems. Karnataka J. Agric. Sci..

[199] Suresha BA, Allolli TB, Patil MG, Hussain SAA (2007). Yield and economics of chilli based intercropping system. Karnataka J. ….

[200] Mahant HDD, Patil SJJ, Bhaleroa PPP, Gaikwad SSS, Kotadia HRR (2012). Economics and land equivalent ratio of different intercrops in banana (Musa paradisiaca L.) cv. GRAND NAINE under drip irrigation. Asian J. Hortic..

[201] Hadidi N, Sharaiha R, Debei HA- (2011). Effect of intercropping on the performance of some summer vegetable crops grown under different row arrangements. Sci. Pap. J. Agron. Ser..

[202] de Lima JSSS, Bezerra Neto F, Zuleide-de-Negreiro M, Cardoso-Ribeiro MC, Barros-Júnior AP (2010). Productive performance of carrot and rocket cultivars in strip-intercropping system and sole crops. Agrocienca.

[203] Degri MM, Samaila AE (2014). Impact of intercropping tomato and maize on the infestation of tomato fruit borer [Helicoverpa armigera (Hubner)]. J. Agric. Crop Res..

[204] Mbah EUU, Ogbodo ENN (2013). Assessment of intercropped sweet corn (Zea mays var. saccharata) and vegetable cowpea (Vigna unguiculata (L.) Walp) using competitive indices in the derived savannah of south-eastern Nigeria. Jounal Biol. Agric. Healthc..

[205] Gebru H, Tsadik K, Tana T, Sadik KWT, Tana T (2015). Evaluation of Tomato (Lycopersicon esculentum Mill) and Maize (Zea mays L.) Intercropping on Growth, Yield and Yield Traits of the Crops in Wolaita Zone, Southern. Ethiopia. J. Biol. Agric. Healthc..

[206] Nthabiseng T, Irvine KM, Moshibudi PM (2015). Response of a maize or dry bean intercrop to maize density and dry bean arrangement under rainfed conditions. Int. J. Agron. Agric. Res..

[207] Mutiga SK, Gohole LS, Auma EO (2011). Agronomic Performance of Collards under Two Intercrops and Varying Nitrogen Application Levels as Assessed Using Land Equivalent Ratios. J. Agric. Sci..

[208] Blazewicz-Wozniak M, Wach D (2011). The effect of intercropping on yielding of root vegetables of Apiaceae family. Acta Sci. Pol. Hortorum Cultus.

[209] Güvenç I, Yildirim E (2005). Intercropping with eggplant for proper utilisation of interspace under greenhouse conditions. Eur. J. Hortic. Sci..

[210] Ghanbari A, Dahmardeh M, Siahsar BA, Ramroudi M (2010). Effect of maize (Zea mays L.) - cowpea (Vigna unguiculata L.) intercropping on light distribution, soil temperature and soil moisture in arid environment. J. Food, Agric. Environ..

[211] Paut R, Garreau L, Ollivier G, Sabatier R, Tchamitchian M (2023). Recherche Data Gouv Repository France.

[212] Lithourgidis AS, Dordas CA, Damalas CA, Vlachostergios DN (2011). Annual intercrops: An alternative pathway for sustainable agriculture. Aust. J. Crop Sci..

[213] Sinclair FL (1999). A general classification of agroforestry practice. Agrofor. Syst..

[214] Nair PKR (1985). Classification of agroforestry systems. Agrofor. Syst..

[215] Mead R, Willey RW (1980). The Concept of a ‘Land Equivalent Ratio” and Advantages in Yields from Intercropping’. Exp. Agric..

[216] McHugh ML (2012). Lessons in biostatistics interrater reliability: the kappa statistic. Biochem. Medica.

[217] Makowski, D., Piraux, F. & Brun, F. *From Experimental Network to Meta-analysis Methods and Applications with R for Agronomic and Environmental Sciences*. *France: Springer Nature B.V* (2019).

[218] Pelzer E, Hombert N, Jeuffroy MH, Makowski D (2014). Meta-analysis of the effect of nitrogen fertilization on annual cereal–legume intercrop production. Agron. J..

[219] Dupraz C (2019). Hi-sAFe: A 3D Agroforestry Model for Integrating Dynamic Tree–Crop Interactions. Sustainability.

[220] Corre-Hellou G, Faure M, Launay M, Brisson N, Crozat Y (2009). Adaptation of the STICS intercrop model to simulate crop growth and N accumulation in pea-barley intercrops. F. Crop. Res..

[221] Cernay C, Pelzer E, Makowski D (2016). Data descriptor: A global experimental dataset for assessing grain legume production. Sci. Data.

[222] Snaydon RW (1991). Replacement or Additive Designs for Competition Studies?. J. Appl. Ecol..

